# Assessing the level of knowledge and available sources of information about hepatitis C infection among HCV-infected Egyptians

**DOI:** 10.1186/s12889-018-5672-6

**Published:** 2018-06-18

**Authors:** Noha Yahia Sultan, Ahmed YacoobMayet, Sinaa Abdulmohsen Alaqeel, Hussain Abdulrahman Al-Omar

**Affiliations:** 1 Department of Microbiology, The National Organization for Drug Control and Research, Cairo, Egypt; 2 Department of Clinical Pharmacy, College of Pharmacy, King Saud University, King Khalid University Hospital, P.O. Box 2457, Riyadh, 11451 Saudi Arabia; 3 Department of Clinical Pharmacy, College of Pharmacy, King Saud University, Riyadh, 11451 Saudi Arabia

**Keywords:** Awareness, Egypt, HCV, Information, Knowledge

## Abstract

**Background:**

Egypt has the largest proportion of hepatitis C virus (HCV) infection worldwide and there is an urgent need to increase community awareness and knowledge about the disease in the country. The main aim of this study was to assess the level of knowledge and awareness about HCV in clinically diagnosed HCV patients in Egypt.

**Methods:**

This was a prospective, cross-sectional study conducted between 1 February 2014 and 30 April 2014 in Cairo, Egypt using validated questionnaire as an instrument for data collection. A structured questionnaire was developed based on similar published surveys. Data collected included demographic characteristics, exposure to the disease, health insurance status, the source of medical information, and knowledge of different routes of transmission; a point was given for each correct answer with a possible score of 0 to 12.

**Results:**

A total of 203 patients took part in this study with a response rate of 90%. Most—142 (70%)—were married, 119 (63%) were unemployed, 127 (62.9%) were aged above 50 years, 88 (45.1%) were living in Cairo, and 45 (22.4%) had a college degree. Half of the participants believed that HCV infection is not transmitted through sex, while 79 (39.9%) did not know that HCV could be transmitted from a mother to her infant during labor. A quarter of participants believed that HCV vaccine is available, and 45 (24.6%) never knew if their treatment was successful. The median knowledge score of HCV infection in the survey was 7.5; 100 (50.3%) participants had ≤ median knowledge score of HCV infection. Logistic regression analysis showed a duration of infection (OR 1.647, CI 1.189–2.82) and the participants who visited physicians when only they felt sick were less likely to have the above median knowledge score (> 7.5) of HCV infection (OR 0.41, 95% CI 0.19–0.87).

**Conclusions:**

Considering the unsatisfactory level of HCV knowledge among infected patients, Egyptian healthcare authorities should organize national awareness campaigns encouraging HCV testing based on educational interventions and activities to improve the level of knowledge. More investment in research is also needed to limit the further growth of the HCV disease burden in Egypt.

**Electronic supplementary material:**

The online version of this article (10.1186/s12889-018-5672-6) contains supplementary material, which is available to authorized users.

## Background

Hepatitis C virus (HCV) infection is one of the major causes of chronic hepatitis and liver disease worldwide and is a serious health issue in many other countries. Worldwide, approximately 130–150 million people are chronically infected with HCV and 500,000 die annually from hepatitis C-related liver diseases [1]. According to the literature, Egypt has the highest rate of HCV infection worldwide with a prevalence that might be as high as 15% of the Egyptian population [1, 2]. Having said that, knowledge and awareness of HCV infection among infected patients have not been well studied. Few studies have been published examining knowledge of HCV in Egypt.

In 2010, Shalaby et al. conducted a questionnaire-based survey in Egypt to determine the prevalence of hepatitis B and C virus infections among barbers and their clients, and to assess the knowledge, attitudes, and practices observable during hair-cutting and shaving. The study showed that there was a reasonable level of knowledge among barbers and their clients about HBV and HCV. However, approximately 25% of the participants did not know that sexual contact and body piercing can transmit the infection; also they believe that hepatitis C vaccine can protect them from infection [3]. Another survey, conducted by Saleh et al. in 2014, included 67 participants and aimed to investigate knowledge, attitudes, and prevention practices related to HCV infection and pesticides use in rural Egypt. The results showed that there was a significant lack of knowledge of HCV infection and its transmission modes [4]. The third study by Chemaitelly et al. in 2013 examined an association between different measures of HCV knowledge and HCV prevalence by analyzing a nationally representative database of 2008 Egyptian Demographic and Health Survey of over 12,000 individuals; They could not establish an association between HCV knowledge and HCV prevalence [5].

In spite of the fact that all three studies indicated the presence of a general lack of knowledge of HCV infections and their modes of transmission, none of them tried to investigate either the sources of available HCV medical information or the level of knowledge and awareness among HCV-infected patients according to age, education level and disease duration. Therefore, the purpose of this study was two-fold: first, to assess HCV knowledge and awareness among patients clinically diagnosed with HCV and, second, to explore available sources of information about HCV.

## Methods

### Study setting

This study was conducted at a charity clinic for specialized liver disease care in Cairo, the capital of Egypt. Cairo was chosen for this study because it has the largest population in Egypt with HCV infection (1,250,000, 8%) [6] compared with all Egypt’s other governorates. Patients were recruited from the Dr. Yassin Abdel Ghaffar Charity Center for Liver Diseases and Research. The center specializes in liver diseases and liver transplantation and provides healthcare services to different social classes free of charge. The center incorporates a hospital with different departments including outpatient clinics, inpatient clinics, an intensive care unit, a radiology department and operating theaters. All the services within this center are provided by trained healthcare professionals such as nurses and physicians, and various hepatology sub-specialists (medical and surgical) including general practitioners, internists, and consultants.

### Study design and inclusion criteria

This was a cross-sectional study conducted in the period from 1 February to 30 April 2014. The study included all HCV patients aged 18 years and over and receiving HCV treatment and/or consultation at the center. Patients were excluded from the study if they were younger than 18 years old, or if they chose to withdraw from the study at any time during the data collection stage, up to the data analysis stage, or if they provided an incomplete questionnaire.

### Participants recruitment process

Three registered nurses (two from the outpatient clinics and one from the inpatient clinics) were trained by the researcher to recruit participants and to distribute the questionnaire according to the study inclusion criteria. They were also available to answer participants’ questions. During recruitment, patients were asked to participate in the survey voluntarily. The study purpose and protocol were explained to all invited patients prior to the interview, and verbal consent was obtained if the patient agreed to participate. To ensure confidentiality, no patients’ names or medical record numbers were required during the interview. If a patient was illiterate and had no companion, the nurse filled in the questionnaire on his/her behalf. All participants were interviewed privately in a designated quiet counseling area to avoid any kind of distraction or disturbance.

### Questionnaire development

A 12-item structured questionnaire was constructed on the basis of similar studies [7, 8]. Because the original questionnaire contained some culturally sensitive questions such as transmission of HCV through tattooing and sharing needles among drug abusers, some of the questions were modified. One reason for doing this was that the estimated number of illicit drug users (IDUs) in Egypt is very low, ranging from 57,000 to 120,000, which less than 0.001%, meaning that omitting the question was unlikely to significantly affect the survey outcome [9]. Another reason was that if any person reports that to health officials that he/she is an IDU sharing needles, that person must be referred to the health care authority. This might have discouraged participants from taking part in the survey. As an alternative, a question about the transmission of HCV through cupping therapy or Hijama was added. Hijama, or cupping therapy, is commonly practiced in Middle Eastern countries including Egypt [10]. It is a traditional Arabic medical therapy where blood is drawn by vacuum from a small skin incision for therapeutic purposes and to relieve pain [10]. Since the original questionnaire was in English, the researcher decided to translate it into Arabic before it was translated back into English and checked by a legal translator.

The questionnaire was revised several times after it was reviewed by individuals from the healthcare field (physicians, pharmacists, and university professors) as well as hepatitis C infected patients, to assure its content validity, accuracy, and clarity of items. Changes to the questionnaire were made based on their feedback and recommendations before completion of the final version. The final version of the questionnaire contained three sections: i) the patient’s socio-demographical data including age, gender, education level, occupation, and resident address to determine our sample geographical distribution; ii) the patient’s current treatment, and drug-related information; and iii) the patient’s knowledge about HCV and its modes of transmission. For the questions measuring knowledge of HCV transmission, a point was given for each correct answer, up to a maximum score of 12; “I don’t know” was considered a wrong answer and given a zero score. A score above the median was suggestive of good knowledge.

A crossover pilot study was conducted using the Arabic translated version; this 12-item questionnaire on HCV knowledge was given to 12 participants for testing. This validation of the questionnaire was done twice with 10 days apart to avoid duplication of the first response. Cronbach’s alpha measures the internal consistency or reliability of a psychometric instrument such as a study questionnaire. A reliability score of 0.60 or higher is required to use a psychometric instrument [11]. Cronbach’s alpha was calculated and results showed a score of 0.74. The corrected item-total correlation coefficient values for all our questions were > 0.3, also indicating that the Arabic version of the HCV knowledge questionnaire had good homogeneity. Institutional Review Board (IRB) approval was obtained from the ethical committee prior to commencing the study.

### Statistical analysis

Descriptive statistics (means, standard deviation, median, counts, and percentages) were used to describe the quantitative and categorical study variables. Pearson’s Chi-square tests (χ^2^) were used to detect whether there were any associations between patients’ socio-demographic and knowledge level. A Binary logistical regression was conducted to identify any association between the independent variables (age group, gender, marital status, education level, employment status, duration of HCV infection, frequency of visiting the physician) and the dependent variable (level of knowledge). The strength of association was calculated and expressed as an estimated value by the adjusted Odds Ratio (OR) with a 95% confidence interval (95% CI). The level of significance was 5%. The Statistical Package for the Social Science (SPSS) version 16.0 for Windows was used for all data analyses.

## Results

A total of 225 patients infected with HCV and attending the charity clinic for specialized liver disease were invited to participate in this study. Of those invited, 203 took part, giving a 90% response rate. Patients’ socio-demographics are summarized in Table 1. The majority of participants—142 (71.3%)—were married, 119 (63%) were unemployed, and 169 (85%) were non-smokers. Moreover, 127 (62.9%) were aged above 50 years, 88 (45%) were living in Cairo and 55% lived in other governorates. There were 115 (56.4%) male patients, 60 (29.9%) were illiterate, while 45 (22.4%) had a college degree. When patients were asked how they discovered their HCV diagnosis, 160 (80.8%) reported that they found out about their HCV status during a routine checkup to assess their general health status. About a third (73, 36.7%) of them had been suffering from HCV infection for more than five years, 21 (10.5%) did not know how long they had the infection, and 105 (55%) were receiving their treatment at the charity institution specializing in liver disease while the rest received their treatment either through governmental institutions (free of charge) or through private insurance (Table 1).

**Table 1:** Socio-demographics of study participants

Socio-demographics	% (n)
Age (*n* = 202)	
≤30 years	14.8 (30)
31–50 years	22.3 (45)
More than 50 years	62.9 (127)
Gender (*n* = 202)	
Female	43.6 (88)
Male	56.4 (114)
Marital Status (*n* = 199)	
Divorced	2.5 (5)
Married	71.4 (142)
Single	7.5 (15)
Widower	18.6 (37)
Education (*n* = 201)	
Illiterate	29.9 (60)
Vocational school/college	34.3 (69)
Primary/secondary school	13.4 (27)
College degree	22.4 (45)
Occupation (*n* = 189)	
Governmental employee	19.5 (37)
Private employee	17.5 (33)
Unemployed	63 (119)
Residency (*n* = 195)	
Cairo	45.1 (88)
Other governorates	54.9 (107)
Are you a smoker (*n* = 199)	
No	84.9 (169)
Yes	15.1 (30)
How did you know you have HCV (*n* = 198)	
Accidently e.g. during routine checkup	80.8 (160)
Upon governmental/non-governmental screening	19.2 (38)
How long you are suffering from HCV (*n* = 199)	
I don’t know	10.5 (21)
Less than 1 year	19.6 (39)
1–5 years	33.2 (66)
More than 5 years	36.7 (73)
Who provides you with treatment of HCV (*n* = 191)	
I am treated in specialist charity institution	55.0 (105)
Governmental insurance	11.5 (22)
Private insurance	33.5 (64)

Table 2 summarizes patients’ treatment and available sources of HCV information, illustrating that the majority of the participants (141, 73.1%) were not frequently receiving information such as how to follow-up once HCV test is positive, how often patient need to test for liver function test and ultrasound to assess the liver status; and where and when to seek treatment. For more than half of the participants, physicians were their primary source of HCV information (102, 53.7%). A third (56, 30.8%) visited their physicians only when they felt sick. They were not always informed about the outcome of their treatment, with 24.6% stating that they did not know whether their HCV treatment had been successful or not and 23.4% presuming that the treatment had been successful because they felt better despite not having seen any laboratory results.

**Table 2:** Questions pertinent to Hepatitis C treatment and of sources of information

Questions	% (n)
Do you frequently receive information about HCV (*n =* 193)	
No	73.1 (141)
Yes	26.9 (52)
If yes, how frequent (*n =* 52)	
6–12 months	72.7 (40)
1–2 years	21.8 (12)
What is your source of HCV information (*n =* 190)	
Physician	53.7 (102)
Mass media	18.4 (35)
Mass media &physician	12.6 (24)
Family member	6.8 (13)
Others (books, internet...etc)	8.5 (16)
How frequently do you visit your physician (*n =* 182)	
Only when I feel sick	30.8 (56)
1 month	19.2 (35)
2 months	11.5 (21)
3 months	27.5 (50)
6 months	11.0 (20)
How will you know if your treatment is successful (*n =* 183)	
I experience less symptoms/feel well	23.5 (43)
My physician informs me with the good lab results	51.9 (95)
I never knew	24.6 (45)
Do you receive the HCV double therapy^a^ (injection and capsule) (*n =* 150)	
No	78.7 (118)
Yes	21.3 (32)

^a^Intravenous interferon alfa-2b and oral ribavirin

The responses to the knowledge questions are summarized in Table 3. Very low proportions of correct responses were observed for Questions 7 and 8 regarding the transmission of HCV infection sexually and from mother to infant (vertical transmission). Only a fifth (38, 19.5%) of participants knew that HCV can be transmitted through sex, while 157 (80.5%) either answered no or did not know about it. Just a third (62 31.3%) of the patients knew that HCV infection can be transmitted vertically during delivery whereas the rest either answered no or did not know about it. And a quarter (50, 25.5%) of the participants mistakenly believed that an HCV vaccine is available. Most of the patients (161, 83%) were aware that HCV can be transmitted by using an infected person’s toothbrush and that it can cause liver damage or cancer (145, 72.9%). Some patients also believed that infection can be transmitted via hand shaking (20, 10.2%), kissing (17, 8.5%) or working (29, 14.5%) with someone who has HCV (Table 3).

**Table 3:** Knowledge of HCV transmission and vaccination

Knowledge question	Participants response		
	Yes % (n)	No % (n)	I don’t know % (n)
Do you believe If someone is infected with HCV, they will most likely carry the virus all their lives (*n* = 190)	46.8 (89)^a^	26.8 (51)	26.4 (50)
Do you believe Infection with HCV can cause the liver damage/ cancer (*n* = 199)	72.9 (145)^a^	8.0 (16)	19.1 (38)
Do you believe someone with HCV can look and feel fine (*n* = 195)	47.7 (93)^a^	40.5 (79)	11.8 (23)
Do you believe HCV can be transmitted by getting a blood transfusion from an infected donor (*n* = 198)	86.4 (171)^a^	5.0 (10)	8.6 (17)
Do you believe HCV can be transmitted by shaking hands with someone who has HCV (*n* = 197)	10.2 (20)	76.6 (151)^a^	13.2 (26)
Do you believe HCV can be transmitted by kissing someone who has HCV (*n* = 199)	8.5 (17)	76.9 (153)^a^	14.6 (29)
Do you believe HCV can be transmitted by having sex with someone who has HCV (*n* = 195)	19.5 (38)^a^	56.4 (110)	24.1 (47)
Do you believe HCV can be transmitted by being born to a woman who had HCV when she gave birth (*n =* 198)	31.3 (62)^a^	28.8 (57)	39.9 (79)
Do you believe HCV can be transmitted by using an infected person’s toothbrush (*n =* 194)	83.0 (161)^a^	10.3 (20)	6.7 (13)
Do you believe HCV can be transmitted by being stuck with a needle or sharp instrument that has HCV–infected blood on it e.g. razors, blades, during cupping therapy (*n =* 199)	87.5 (174)^a^	6.5 (13)	6.0 (12)
Do you believe HCV can be transmitted by working with someone who has HCV (*n =* 200)	14.5 (29)	65.0 (130)^a^	20.5 (41)
Do you believe there is vaccination for HCV (*n =* 198)	25.3 (50)	43.5 (86)^a^	31.2 (62)

^a^Correct answer

**Fig. 1:**
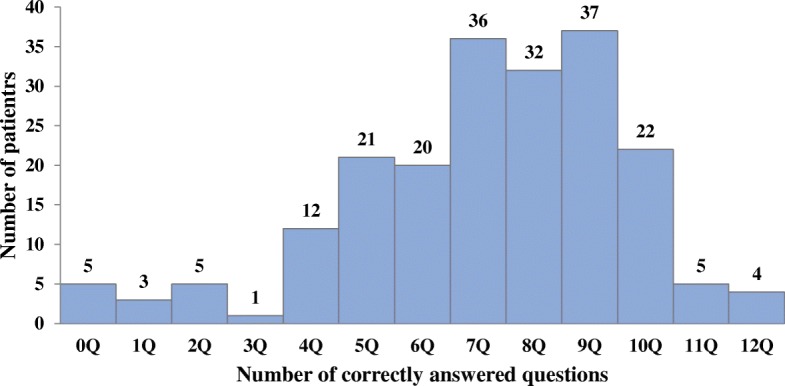
The distribution of knowledge scores

Overall, the median knowledge score of HCV infection in the survey was 7.5 (range 0–12); 100 (50.3%) participants had an equal to or below median knowledge score of HCV infection whereas 99 (49.7%) had an above median knowledge score. The distribution of knowledge scores can be found in Fig. 1. Cronbach’s alpha coefficient for the 12-item scale was 0.742.

We found a significant association between education levels, duration of HCV infection, the frequency of visiting the physicians and knowledge of HCV infection (Table 4). The participants with a lower education level (less than a college degree) were at least 2 times more likely to have below or equal (≤) median knowledge score of HCV infection than those with college graduates (OR 2.1, 95% CI 1.05–4.19). Also, participants with HCV infection for less than a year were at least 3 times more likely to have ≤ median knowledge score (≤7.5) of HCV infection than those who had had the infection for more than 5 years (OR 3.4, 95% CI 1.50–7.72). The participants who visited physicians when only they felt sick were less likely to have the above median knowledge score (> 7.5) of HCV infection (OR 0.5, 95% CI 0.26–0.98) (Table 4).

**Table 4:** Association between demographic characteristics and knowledge level

Background characteristics		Total (n)	Knowledge group %(n)		OR (95% CI)
			Low knowledge	High Knowledge	
Age group	< 50 years	74	58.1(43)	41.9(31)	1.65(.0.93,2.96)
	> 50 years	125	45.6(57)	54.4(68)	1
Marital status	Single	15	27.0(4)	73.3(11)	0.35(0.11,5.16)
	Widow/Divorced	41	58.5(24)	41.5(17)	1.37(0.68,2.77)
	Married	140	50.7(71)	49.3(69)	1
Education level	Others	154	54.5(84)	45.5(70)	2.1(1.05,4.19)
	College	44	36.4(16)	63.6(28)	1
Gender	Female	86	57.0(49)	43.0(37)	1.61(0.91,2.83)
	Male	113	45.1(51)	54.9(62)	1
Employment status	Unemployed	118	54.2(64)	45.8(54)	1.41(0.78,2.58)
	Employee	68	45.6(31)	54.4(37)	1
Duration of HCV infection	I don’t know	20	65.0(13)	35.0(7)	3.16 (1.16,8.63)
	< 1 year	39	66.7(26)	33.3(13)	3.41 (1.50,7.72)
	1–5 years	66	50.0(33)	50.0(33)	1.70 (0.86,3.35)
	> 5 years	73	37.0(27)	63.0(46)	1
Frequency of visiting the physician	Only when feel sick	56	37.5(21)	62.5(35)	0.50 (0.26,0.98)
	Scheduled visit (1–6) months	123	58.3(67)	41.7(56)	1

Logistic regression analysis showed an independent association between the duration of HCV infection, scheduled visit to the physicians; and ≤ median knowledge score of HCV infection. The patients with HCV infection for less than a year were at least 1.5 times more likely to have ≤ median knowledge score (≤7.5) of HCV infection compared to those who had had the infection for more than 5 years (OR 1.56, 95% CI 1.20–2.45); and the participants who visited physicians when only they felt sick were less likely to have the above median knowledge score (> 7.5) of HCV infection (OR 0.41, 95% CI 0.19–0.87) after adjusting for other variables (age group, marital status, gender, education level, employment status).

The level of knowledge questions were tested for differences in proportion with a correct response by age, education level, duration of HCV infection, the frequency of visits to physicians, residency status, and frequency of receiving information about HCV infection. Significant differences were observed in all domains (Additional file 1: Table S1 and Table S2).

## Discussion

Hepatitis C is a major and growing health issue in Egypt, which has the largest proportion of HCV infection in the world; this high prevalence brings negative clinical and economic consequences on patients, their families, and healthcare systems. Several studies have attempted to identify the level of knowledge and awareness about HCV infection among the general population in Egypt and worldwide but not among individuals who are infected with the virus [3, 7, 12]. This study has attempted to assess the level of knowledge and awareness of hepatitis C infection among HCV positive patients using a psychometric instrument.

The present study revealed that correct knowledge of the modes of transmission of HCV among Egyptian HCV-infected patients was unsatisfactory in most of the participants. The proportions of correct responses to the knowledge questions among HCV-infected patients varied widely, ranging from 19.5 to 87.5% with various knowledge gaps and misbeliefs about HCV modes of transmission in areas such as vertical transmission and transmission through hand-shaking, kissing or working with someone who has HCV. This finding supports the results from Chemaitelly et al., who suggested that a substantial proportion of the general population in Egypt had poor levels of knowledge in relation to HCV modes of transmission. They found that 61% of participants knew HCV could be transmitted through blood transfusions, 51% knew about transmission through sharing of unclean needles, the majority (94%) were not aware of mother-to-child transmission, and 14% believed that HCV can spread through casual physical contact [5]. Similarly, the results from Saleh et al.’s study revealed that 22% of participants did not know what causes HCV infection, 81% mentioned incorrect modes of transmission, and 45% did not know the disease manifestations [4]. However, the findings of this current study were inconsistent with those of Shalaby et al., who claimed that the level of knowledge among participants was relatively high, with over 80% positive responses for most questions, especially regarding modes of transmission, although approximately 25% of the participants did not know that sexual contact and body piercing can transmit the infection and believed that hepatitis C vaccine can protect them from disease [3]. It should be taken into consideration, though, that no attempt was made by Shalaby et al. to distinguish between the level of knowledge about HBV infection and that about HCV infection, i.e. participants might have had a better understanding of HBV infection but not known so much about HCV infection, and vice versa.

One of the most remarkable results of this study was that the majority of the participants (80.8%) had been unaware of their HCV infection, only finding out about it accidentally during a routine checkup to assess their general state of health. It is particularly important since HCV 70 to 85% of HCV-infected individuals are asymptomatic because of the slow onset of the disease; can be only uncovered by serological testing [8]. Similarly, a previous study reported that 72% of its participants had been unaware of their HCV infection status [13]. A survey conducted in the United States by the National Health and Nutrition Examination Survey (NHANES) from 2001 through 2008 showed that almost 50% of its participants who were previously tested positive for HCV infection not aware of their infection status in a follow-up survey [8].Educational interventions significantly improved knowledge and increased acceptability of testing, increase in the willingness of patients with HCV to seek treatment, to follow up on the disease, and to prevent transmission to others [14]. Sequentially, this is likely to decrease the overall infection rate and the mortality rate as well as the costs arising from the treatment of liver cirrhosis and its complications [14]. Therefore, Egyptian healthcare authority should adopt or establish guidelines or recommendations similar to those in American Association for the Study of Liver Diseases (AASLD) and the Infectious Diseases Society of America (IDSA) for testing, managing, and treating Hepatitis C patients [15] to test and screen high risk individuals, whether they fall under risk behaviors or risk exposures categories, for HCV infection and test them for early detection of infection and prioritize the treatment.

Three-quarters of our patients were not frequently receiving information on how to follow up once HCV is detected, how often to do a liver function test and an ultrasound to assess liver status and the presence of any liver cancer, and where and when to seek HCV treatment. Half of them received information about their disease from their physicians. In contrast, Shalaby et al. showed that friends and relatives (47.9%), followed by television (43%), newspapers (36.7%), and doctors (30%) were the main sources of information [3]; Chemaitelly et al. showed that the media constituted the main sources of HCV knowledge [5]; and the majority of respondents in Saleh et al.’s study considered physicians (82%) and television (78%) to be the main effective sources of information [4]. These conflicting findings might suggest that public receive inaccurate, incomplete or confusing information depending on the source from which they seek it. Egyptian healthcare authority needs comprehensive plans that address increase knowledge, awareness, and prevent HCV infection among the general public and offer care and treatment for HCV infected patients. In 2012, the Egyptian Ministry of Health and Population (MOHP) had established 23 hepatitis treatment facilities to provide service to 190,000 chronic HCV infected patients. In spite of such attempt by MOHP, the country is facing a continuing hepatitis C epidemic due to:i) lack of healthcare workers with specific training or expertise in infection control; ii) lack of formal infection control programs in most facilities; and iii) poor understanding among healthcare workers regarding standard precautions for infection control [16]. This was evident from the results of our study, which showed that almost 52% of participants were informed by their treating physician about their successful therapy and that 25% of participants mistakenly thought that an HCV vaccine is available. This is in line with the results from Shalaby et al.’s study, which found that around 25% claimed to know about a protective vaccine for HCV [3].

Logistic regression of our study identified the patients who had HCV infection for less than a year and the patients who visit physicians when they felt sick had ≤ median knowledge score of HCV infection after adjusting for other dependent variables. Therefore, strategies aiming to limit the spread of HCV infections, such as screening programs and educational campaigns, should consider in recently diagnosed patients and those with a low educational level to encourage them to have scheduled physician visit in the strategic plan. Moreover, current and future strategies, tools, and resources to limit HCV infection spread should be evaluated through implementation research to assure their efficiency in disseminating key messages for individuals with low level of knowledge.

This study has several strengths compared with others. First, its sample included clinically diagnosed HCV positive patients with different socio-demographic backgrounds in contrast to many studies that focus on participants from the general public. Second, the recruitment site was a specialized center for liver diseases, which makes the findings likely more valid with regard to the levels of knowledge and information recorded, especially considering that the majority of participants in this study indicated that their physician was their primary source of information. Third, this study not only examined knowledge of the modes of HCV transmission, but also looked at the socio-demographic characteristics of patients with HCV infection. Fourth, the Arabic translated questionnaire that was used had good reliability and homogeneity compared with other instruments.

As with all studies, there are a number of limitations to consider in terms of the research design and data collection method, including the use of a quantitative closed-ended questionnaire rather than a qualitative focus group or semi-structured interviews with open-ended questions, which would have given more insight and in-depth understanding about participants’ awareness and knowledge about HCV infection. Additionally, the study was limited to a single center with a relatively small sample size and short duration.

## Conclusions

Considering the relatively low level of HCV knowledge among HCV-infected patients and the high prevalence of the disease in Egypt, this study suggests that more intensive efforts are needed to enforce the provision of education for patients and individuals by healthcare professionals. Additionally, the Egyptian healthcare authority needs to launch national awareness campaigns and activities to disseminate accurate information about HCV among society, with a focus on subjects where there is a gap or weakness in knowledge. There is also room for conducting further research on patients’ and healthcare professionals’ behavior, in order to tackle the escalating disease burden of HCV. Other innovative approaches such as social media and networking to disseminate key information on HCV infection from healthcare authorities should be explored.

## Additional file

Additional file 1: **Table S1.** Impact of age, education background, and duration of HCV infection on providing correct answer on knowledge questionnaire. **Table S2.** Impact of frequent physician visits, information received, and residency on providing correct answer on knowledge questionnaire. (DOCX 25 kb)

## Abbreviations

AASLD: American Association for the Study of Liver Diseases
HCV: Hepatitis C Virus
IDSA: Infectious Diseases Society of America
IDUs: Illicit Drug Users
IRB: Institutional Review Board
MOHP: Egyptian Ministry of Health and Population
SPSS: Statistical Package for the Social Science
