# Supplementary material for: Assessing the level of knowledge and available sources of information about hepatitis C infection among HCV-infected Egyptians
Source: BMC Public Health. 2018 Jun 18;18:747. doi: 10.1186/s12889-018-5672-6 (PMC6007060; doi:10.1186/s12889-018-5672-6)
Supplement: Supplementary file 1 — Table S1. Impact of age, education background, and duration of HCV infection on providing correct answer on knowledge questionnaire. Table S2. Impact of frequent physician visits, information received, and residency on providing correct answer on knowledge questionnaire. (DOCX 25 kb) [file 12889_2018_5672_MOESM1_ESM.docx]

**Supplementary files results**

With respect to age, for Question 1 (If someone is infected with HCV, they will most likely carry the virus all their lives), we found that a significantly higher proportion of patients with age ≥ 50 (54.2%) answered correctly (p<0.001). For Question 9 (HCV can be transmitted by kissing) and Questions 10 (HCV can be transmitted by shaking hands with someone who has HCV), those with age 31–50 were significantly less likely to respond correctly (57.8% and 61.4%) with p<0.01. For Question 12, related to vertical transmission, the number of patients who answered correctly was significantly higher in the age group ≤ 30 (62.1%, p<0.01) (Supplement 1).

Regarding education level, we found that the responses of illiterate patients with HCV infection were significantly poorer (p<0.001) to Questions 6 to 11 concerning vertical transmission (Q6) and HCV transmission by using an HCV-infected person’s toothbrush (Q7), being stuck with a needle or sharp instrument with HCV-infected blood on it (Q8), kissing (Q9) or shaking hands (Q10) with an HCV-infected person, or working with someone who has HCV infection (Q11).

With respect to duration of HCV infection, the patients who had had HCV infection for less than a year gave significantly poorer responses (p<0.01) to Question 2 about whether HCV infection can cause liver cancer or damage and to Questions 9 and 10 concerning HCV transmission via kissing or shaking hands with HCV-infected patients (Table S1).

With respect to frequency of visiting physicians, we found that patients who visit more frequently (every 1 to 2 months) gave significantly poorer responses (p<0.01) to Question 7 about becoming infected through using the toothbrush of an infected patient and to Questions 9 and 10 about HCV transmission via kissing or shaking hands with HCV-infected patients. When asked, “Do you frequently receive information about HCV?”, a significant proportion of the patients who answered “yes” had better knowledge on Questions 4 (HCV transmission via infected blood), 7 (transmission via an infected patient’s toothbrush), 9 and 10 (HCV transmission via kissing or shaking hands), and 11 (HCV transmission from working with HCV-infected patients) with p<0.01 (Table S2).

**Table S1: Impact of age, education background, and duration of HCV infection on providing correct answer on knowledge questionnaire**

| **Knowledge Questions** | **Age group (years)**  **(with correct answer)**  **n=202** | | | **Education levels**  **(with correct answer)**  **n=201** | | | | **How long (yrs) are you suffering from HCV?**  **(with correct answer) *n=178** | | |
| --- | --- | --- | --- | --- | --- | --- | --- | --- | --- | --- |
|  | **≤30** | **31-50** | **>50** | **College** | **Illiterate** | **Primary & Secondary** | **Vocational school/college** | **<1 yr** | **1-5 yrs** | **>5yrs** |
| 1.Do you believe If someone is infected with HCV, they will most likely carry the virus all their lives (n=190) | 34.5† | 33.3 | 54.2 | 58.5 | 43.1 | 48.0 | 41.5 | 42.9 | 43.1 | 50.0 |
| 2.Do you believe Infection with HCV can cause the liver damage/ cancer (n=199) | 64.3 | 68.9 | 76.0 | 90.9† | 59.3 | 63.0 | 76.1 | 65.8† | 66.7 | 87.7 |
| 3.Do you believe someone with HCV can look and feel fine (n=195) | 58.6 | 36.4 | 48.8 | 61.9 | 35.1 | 50.0 | 47.1 | 43.6 | 46.2 | 54.3 |
| 4.Do you believe HCV can be transmitted by getting a blood transfusion from an infected donor (n=198) | 86.2 | 84.1 | 87.1 | 93.2 | 79.3 | 92.6 | 85.1 | 89.5 | 83.3 | 88.9 |
| 5.Do you believe HCV can be transmitted by having sex with someone who has HCV (n=195) | 17.9 | 17.8 | 20.7 | 20.9 | 13.8 | 19.2 | 24.2 | 23.7 | 13.8 | 23.6 |
| 6.Do you believe HCV can be transmitted by being born to a woman who had HCV when she gave birth (n=198) | 37.9 | 33.3 | 36.0 | 30.2†† | 16.9 | 19.2 | 48.5 | 28.2 | 28.1 | 37.0 |
| 7.Do you believe HCV can be transmitted by using an infected person’s toothbrush (n=194) | 77.8 | 85.4 | 83.2 | 95.3†† | 67.2 | 84.6 | 89.2 | 81.1 | 80.6 | 89.0 |
| 8.Do you believe HCV can be transmitted by being stuck with a needle or sharp instrument that has HCV–infected blood on it e.g. razors, blades, during cupping therapy (n=199) | 89.7 | 84.4 | 88.7 | 90.9†† | 72.4 | 92.6 | 95.6 | 87.2 | 86.2 | 91.8 |
| 9.Do you believe HCV can be transmitted by shaking hands with someone who has HCV (n=197) | 82.1† | 57.8 | 82.1 | 90.9††† | 57.6 | 80.8 | 81.8 | 63.2† | 76.9 | 84.7 |
| 10.Do you believe HCV can be transmitted by kissing someone who has HCV (n=199) | 82.8† | 61.4 | 80.8 | 81.8† | 61.0 | 81.5 | 85.1 | 57.9† | 81.8 | 86.3 |
| 11.Do you believe HCV can be transmitted by working with someone who has HCV (n=200) | 75.9 | 60.0 | 64.8 | 84.1† | 50.8 | 55.6 | 67.6 | 61.5 | 68.2 | 68.5 |
| 12.Do you believe there is vaccination for HCV (n=198) | 62.1† | 28.9 | 43.9 | 47.7 | 40.7 | 50.0 | 38.8 | 35.9 | 49.2 | 45.2 |

*21 Patients who did not know their how long they were infected with HCV were excluded from analysis.

† P<0.05, †† P< 0,001, ††† P< 0.0001

**Table S2: Impact of frequent physician visits, information received, and residency on providing correct answer on knowledge questionnaire**

| **Hepatitis Knowledge Questions** | **Frequency of visiting the physicians**  **(with correct answer)**  **n=182** | | | **Residency**  **(with correct answer)**  **n=195** | | **Do you frequently receive information about HCV?**  **(with correct answer) n=193** | |
| --- | --- | --- | --- | --- | --- | --- | --- |
|  | **Only when I feel sick** | **1 -2 months** | **3 – 6**  **months** | **Cairo** | **Other governorates** | **Yes** | **No** |
| 1.Do you believe If someone is infected with HCV, they will most likely carry the virus all their lives (n=190) | 63.6 | 34.7 | 42.4 | 48.1 | 43.2 | 45.0 | 49.2 |
| 2.Do you believe Infection with HCV can cause the liver damage/ cancer (n=199) | 75.0 | 68.5 | 77.9 | 72.9 | 72.8 | 71.9 | 72.7 |
| 3.Do you believe someone with HCV can look and feel fine (n=195) | 54.5 | 38.5 | 53.7 | 51.3 | 45.2 | 54.8 | 44.4 |
| 4.Do you believe HCV can be transmitted by getting a blood transfusion from an infected donor (n=198) | 92.7 | 79.2 | 89.9 | 88.2 | 85.0 | 93.8† | 81.9 |
| 5.Do you believe HCV can be transmitted by having sex with someone who has HCV (n=195) | 27.3 | 18.5 | 18.2 | 20.2 | 18.9 | 26.7 | 17.2 |
| 6.Do you believe HCV can be transmitted by being born to a woman who had HCV when she gave birth (n=198) | 37.5 | 25.9 | 28.4 | 30.6 | 31.9 | 35.5 | 28.7 |
| 7.Do you believe HCV can be transmitted by using an infected person’s toothbrush (n=194) | 96.3† | 71.7 | 85.1 | 83.1 | 82.9 | 92.1† | 78.2 |
| 8.Do you believe HCV can be transmitted by being stuck with a needle or sharp instrument that has HCV–infected blood on it e.g. razors, blades, during cupping therapy (n=199) | 98.2† | 77.8 | 86.8 | 88.1 | 87.0 | 90.5 | 85.3 |
| 9.Do you believe HCV can be transmitted by shaking hands with someone who has HCV (n=197) | 78.2† | 63.5 | 88.4 | 79..8 | 74.3 | 87.3† | 70.1 |
| 10.Do you believe HCV can be transmitted by kissing someone who has HCV (n=199) | 78.2 | 70.4 | 84.1 | 77.6 | 76.3 | 84.4† | 71.9 |
| 11.Do you believe HCV can be transmitted by working with someone who has HCV (n=200) | 55.4 | 64.8 | 72.5 | 68.2 | 62.6 | 75.0† | 58.9 |
| 12.Do you believe there is vaccination for HCV (n=198) | 41.1 | 40.7 | 46.3 | 47.1 | 40.7 | 50.0 | 39.5 |

† P<0.05, †† P< 0,001, ††† P< 0.0001

**Supplementary Files Discussion**

The present study found higher proportions of incorrect answers among patients aged younger than 50 years, patients who did not pursue their college education, patients who had been aware of their HCV infection for less than five years, patients who visited physicians frequently, patients from rural governates, and patients who rarely received information about HCV. This is consistent with the findings from Chemaitelly et al., who concluded that having a lower educational level and being a resident of a rural area are the main risk factors for HCV infection [5]. Therefore, strategies aiming to limit the spread of HCV infections, such as screening programs and educational campaigns, should consider recently diagnosed patients, those with a low educational level or a young age, residents of rural communities and those who rarely visit their physician visits in the strategy plan. Moreover, current and future strategies, tools, and resources to limit HCV infection spread should be evaluated through implementation research to assure their efficiency in disseminating key messages for individuals with low level of knowledge.
